# High resolution melting assay as a reliable method for diagnosing drug-resistant TB cases: a systematic review and meta-analysis

**DOI:** 10.1186/s12879-021-06708-1

**Published:** 2021-09-22

**Authors:** Masoud Keikha, Mohsen Karbalaei

**Affiliations:** 1 Department of Microbiology and Virology, Faculty of Medicine, Mashhad University of Medical Sciences, Mashhad, Iran; 2 Student Research Committee, Mashhad University of Medical Sciences, Mashhad, Iran; 3 Department of Microbiology and Virology, School of Medicine, Jiroft University of Medical Sciences, Jiroft, Iran

**Keywords:** Drug-resistant tuberculosis, High resolution melting, *Mycobacterium tuberculosis*

## Abstract

**Background:**

Tuberculosis (TB) is one of the most contagious infectious diseases worldwide. Currently, drug-resistant *Mycobacterium tuberculosis* (*Mtb*) isolates are considered as one of the main challenges in the global TB control strategy. Rapid detection of resistant strains effectively reduces morbidity and mortality of world’s population. Although both culture and conventional antibiotic susceptibility testing are time-consuming, recent studies have shown that high resolution melting (HRM) assay can be used to determine the types of antibiotic resistance. In the present meta-analysis, we evaluated the discriminative power of HRM in detecting all drug-resistance cases of TB.

**Methods:**

A systematic search was performed using databases such as Cochrane Library, Scopus, PubMed, Web of Science, and Google Scholar. Related studies on the effect of HRM in the diagnosis of drug-resistant (DR) TB cases were retrieved by April 2021. We used Meta-Disc software to evaluate the pooled diagnostic sensitivity and specificity of HRM for the detection of each type of drug-resistant cases. Finally, diagnostic value of HRM was characterized by summary receiver operating characteristic (SROC) curve and the area under the curve (AUC) method.

**Results:**

Overall 47 studies (4,732 *Mtb* isolates) met our criteria and were included in the present meta-analysis. Sensitivity, specificity, and AUC of HRM were measured for antibiotics such as isoniazid (93%, 98%, 0.987), rifampin (94%, 97%, 0963), ethambutol (82%, 87%, 0.728), streptomycin (82%, 95%, 0.957), pyrazinamide (72%, 84%, 0.845), fluoroquinolones (86%, 99%, 0.997), MDR-TB (90%, 98%, 0.989), and pan-drug-resistant TB (89%, 95%, 0.973).

**Conclusions:**

The HRM assay has high accuracy for the identification of drug-resistant TB, particularly firs-line anti-TB drugs. Therefore, this method is considered as an alternative option for the rapid diagnosis of DR-TB cases. However, due to heterogeneity of included studies, the results of HRM assays should be interpreted based on conventional drug susceptibility testing.

## Background

A century after the discovery of *Mycobacterium tuberculosis* (*Mtb*) as the etiological agent of tuberculosis (TB) by Robert Koch, the disease is still one of the leading causes of death (after AIDS) worldwide [1, 2]. According to the World Health Organization (WHO) report in 2020, approximately 10 million (range, 8.9–11.0 million) people became infected with *Mtb* in 2019; of these, approximately 1.2 million (range, 1.1–1.3 million) deaths occurred among HIV-negative people [3]. Furthermore 208,000 deaths (range, 177,000–242,000) were related to HIV-positive individuals [4]. According to the use of comprehensive treatment programs, statistics show that the number of TB deaths among HIV-positive and HIV-negative people fell by 31% and 69% between 2000 and 2019, respectively [4]. Drug-resistant tuberculosis (DR-TB) is a global concern for TB control programs, and close to half a million people have been diagnosed with rifampicin-resistant TB (RR-TB); of which 78% had MDR-TB (resistant to both rifampicin and isoniazid). Three countries, India (27%), China (14%), and Russian Federation (8%) had the largest share of the global MDR-TB burden, respectively [4, 5]. Therefore, prompt diagnosis and appropriate treatment of both TB and DR-TB cases are considered as key factors in reducing mortality from this disease [6, 7]. According to WHO guidelines, diagnosis of both resistance and susceptible isolates requires methods such as molecular techniques, cultivation, sequencing, and bacteriological confirmation; although methods e.g. culture (in LJ, 7H10, 7H11 and MGIT media) and phenotypic drug susceptibility testing (DST) are considered as gold standard methods for diagnosis of this bacterium, however, these methods are time-consuming, laborious, and sometimes ambiguous results are obtained [4, 8]. Therefore, reducing the detection time leads to reduce the risk of transmission of resistant strains [9]. In recent years, several rapid methods for diagnosing TB or DR-TB isolates such as PCR, real-time PCR, line probe assay (LPA), Xpert MTB/RIF assay, and BACTEC MGIT 960 liquid culture have been introduced [8]. Xpert MTB/RIF assay is applicable in high-prevalence and low-income settings; although this method has recently been approved by WHO for rapid detection of both *Mtb* and RR-TB isolates, it has several disadvantages, for example, it is only used for rifampin and is not affordable in all regions of developing countries [8–11]. BACTEC system is based on the generation of radioactive CO_2_ from substrate palmitic acid; this system is high specific and helpful in distinguishing *Mtb* from other mycobacteria, as well as is used as a comparative method versus DST. In general, in this system growth can be detected in 5–10 days; however, the detection time of bacteria in this method is faster than the conventional culture method (more than two weeks), but it takes more time to detect than molecular methods [12]. Overall, molecular methods are more rapid compared to other methods, nevertheless, these methods are costly, and also require standardization and a multi-step process; moreover, the use of these methods increases the risk of cross-contamination and misdiagnosis [13]. High resolution melting (HRM) assay is a new method to detect point mutations, single nucleotide polymorphism (SNP), and internal tandem duplications [14, 15]. HRM technique is based on differences in the melting profiles of test and reference DNAs; in this method, using real-time PCR assay, first, immunofluorescent dyes bind to double-stranded DNA, and during the denaturation step of PCR amplicons, differences in melting curves indicate that a mutation occurs in the test DNAs compared to reference DNA [16]. The recent method is relatively inexpensive and requires only unlabeled primers and dsDNA binding dyes; the process of detection is too fast, and due to the reaction in a closed tube, the risk of contamination is low; unlike other molecular methods, this method has no post-PCR process [17, 18]. To date, various studies have examined the sensitivity and specificity of HRM, and the aim of this meta-analysis was to determine the overall accuracy of HRM in identifying DR-TB cases.

## Methods

### Literature search strategy

The systematic review was performed based on preferred reporting items for systematic reviews and meta-analysis (PRISMA) guideline [19]. At the first, a comprehensive literature search‏ ‏was‏ ‏conducted‏ ‏using global databases such as‏ ‏Web of Science, PubMed, Scopus, Cochrane Library, and Google scholar. Relevant studies were retrieved according to keywords such as “*Mycobacterium tuberculosis*”, “Tuberculosis”, “TB”, “Drug-resistant TB”, “High resolution melting”, “Specificity”, and “Sensitivity”. Studies were selected regardless of publication date, and we also evaluated the bibliography of all candidate articles to identify duplicate studies.

### Study screening and selection

To determine the eligibility of studies, title, abstract, and full text of potential studies were evaluated. This process was conducted by two authors separately, and disagreements were resolved through discussion. Our inclusion criteria included: (1) original articles on HRM value for diagnosis of DR-TB; (2) evaluated studies by reference tests (antibiogram, BACTEC MGIT 960 system, sequencing) and HRM; (3) studies containing information such as true positive (TP), false positive (FP), true negative (TN), and false negative (FN); (4) English studies. In the present study, excluded criteria were including: (I) congress abstracts, letters, and review articles; (II) articles with unclear results and insufficient data; (III) studies on extrapulmonary TB; (IV) non-English studies. Finally, 24 articles met our included criteria [20–43]. ‏

### Data extraction and quality assessment

In this step, we used the Newcastle–Ottawa Scale (NOS) to assess the quality of included studies. Information such as first author, publication year, country, subjects, number of *Mtb* (resistant and susceptible) isolates, studied genes, and number of TP/FP/TN/FN for each participant are listed in Table 1.

**Table 1:** Characteristic of included studies

Author	Year	Country	Subjects	R/S	Gene	TP (n)	FP (n)	FN (n)	TN (n)	NOS	Refs.
Pietzka	2009	Austria	Multidrug-resistant TB	49/19	*rpoB*	44	2	5	17	8	[18]
Choi	2010	Korea	Isoniazid resistance Rifampicin resistance	100/117 73/124	*katG*, *inhA* *rpoB*	90 72	0 0	10 1	117 124	10	[19]
Ong	2010	Singapore	Isoniazid resistance Rifampicin resistance	53/6 28/31	*katG* and *mab-inhA* *rpoB*	52 25	1 0	1 3	5 31	8	[20]
Ramirez	2010	United States	Multidrug-resistant TB	148/104	*katG*, *inhA* *rpoB*	126	2	22	102	8	[21]
Wang	2011	China	Streptomycin resistance	30/0	*rpsL*	21	0	9	0	5	[22]
Chen	2011	Australia	Isoniazid resistance Rifampicin resistance Ofloxacin resistance	69/46 54/61 41/74	*katG* and *mab-inhA* *rpoB* *gyrA*	67 51 41	0 1 1	2 3 0	48 60 73	9	[23]
Lee	2012	Singapore	Fluoroquinolone and Streptomycin resistance	25/28 48/14	*gyrA* *rpsL*	19 42	0 0	6 6	28 14	7	[24]
Yadav	2012	India	Isoniazid resistance Rifampicin resistance Streptomycin resistance	35/20 29/20 34/20	*katG* *rpoB* *rpsL*	30 27 21	2 0 0	5 2 13	18 20 20	9	[25]
Nagai	2013	Japan	Isoniazid resistance Rifampicin resistance Ethambutol resistance Streptomycin resistance	12/15 10/17 8/19 11/16	*katG*, *mab-inhA* *rpoB* *embB* *rpsL*, *rrs*	11 10 8 11	0 2 0 1	1 0 0 0	15 15 19 15	10	[26]
Nour	2013	Egypt	Isoniazid resistance Rifampicin resistance	20/10 13/17	*katG*, *rpoB*	17 12	0 0	3 1	10 17	6	[27]
Haeili	2014	Iran	Isoniazid resistance Rifampicin resistance	21/54 20/54	*katG* *rpoB*	18 19	0 0	3 1	54 54	8	[28]
Pholwat	2014	United States	Pyrazinamide resistance	55/41	*pncA*	34	7	21	34	8	[29]
Malhotra	2015	India	Rifampicin resistance	103/116	*rpoB*	93	3	10	113	9	[30]
Pholwat	2015	United states	Drug-resistant TB	186/41 161/58 80/123 107/304 40/180 56/57	*inhA* or *katG* *rpoB* *embB* *rpsL*, *rrs*, *eis* *gyrA-gyrB* *pncA*	174 153 62 89 31 49	0 11 35 18 3 2	12 8 18 18 9 7	41 47 88 286 177 55	10	[31]
Osman	2016	Africa	Pyrazinamide resistance	29/66	*pncA*	14	17	15	49	7	[32]
Galarza	2016	Peru	Multidrug-resistant TB Isoniazid resistance Rifampicin resistance	78/89 78/89 78/89	*katG*, *inhA* *rpoB*	77 77 77	2 2 0	1 1 1	87 87 89	8	[33]
Anthwal	2017	India	Isoniazid resistance Rifampicin resistance	21/78 11/88	*katG*, *inhA* *rpoB*	18 10	0 0	3 1	88 88	9	[34]
Rezaei	2017	Iran	Ethambutol resistance Streptomycin resistance	21/55 25/51	*embB* *rrs*, *rpsL*	19 22	2 0	2 3	53 51	6	[35]
Sirous	2018	Iran	Isoniazid resistance Rifampicin resistance Ofloxacin resistance	16/20 18/20 5/20	*katG* and *mab-inhA* *rpoB* *gyrA*	14 15 4	0 0 0	2 3 1	20 20 20	10	[36]
Negi	2018	India	Multidrug-resistant TB	94/49	*katG*, *rpoB*	85	0	9	49	8	[37]
Filipenko	2019	Russia	Pyrazinamide resistance	38/20	*pncA*	31	3	7	17	7	[38]
Arefzadeh	2020	Iran	Rifampicin resistance	5/75	*rpoB*	5	8	0	67	8	[39]
Anukool	2020	Thailand	Isoniazid resistance Rifampicin resistance	34/69 37/70	*katG*, *inhA* *rpoB*	33 31	4 1	1 6	65 69	9	[40]
Wang	2020	China	Ethambutol resistance	59/163	*embB*	49	9	10	154	7	[41]

### Statistical analysis

The accuracy and reliability of HRM method were measured using indexes such as sensitivity, specificity, and diagnostic odds ratio (DOR) with 95% confidence interval (CI). Subsequently, we employed summary receiver operating characteristic (SROC) curve to measure the area under the curve (AUC) [44]. In addition, Chi-squared and I-squared tests (p < 0.01 or *I*^*2*^ > 50%) were used to measure heterogeneity between studies. Based on significant heterogeneity between studies, we conducted a pooled analysis (fixed-effects model or random-effects model). Furthermore‏,‏ subgroup analysis was performed‏ ‏individually‏ ‏to assess the accuracy of HRM assay for two types of DR-TB including, mono-resistant TB (rifampin, isoniazid, ethambutol, streptomycin, pyrazinamide, or fluoroquinolone) and MDR-TB‏.‏ All statistical‏ ‏tests‏ ‏were two-sided with a significant cut-off (p < 0.05), and were fulfilled by Meta-DiSc software.

## Results

### Characterization of included studies

**Fig. 1:**
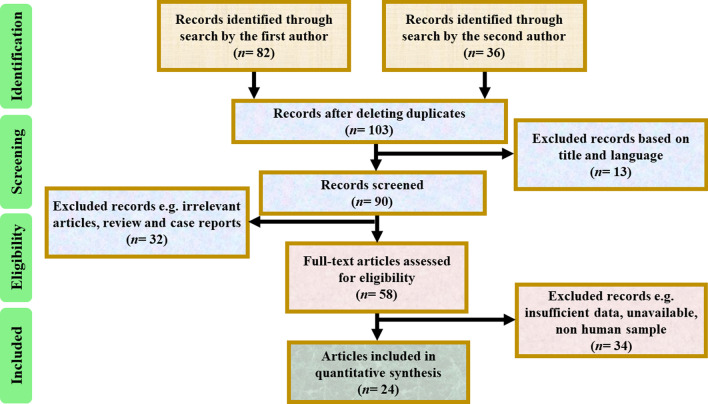
Flowchart of the selection of excluded and included studies

After an initial evaluation of the retrieved articles, finally 24 studies were accordance with our criteria (Fig. 1). Included studies had been published in Austria, Korea, Singapore, United States, China, India, Iran, Japan, Egypt, Africa, Peru, Russia, and Thailand during 2009–2020. In this study, data from 4732 *Mtb* isolates (both drug-resistant and drug-susceptible strains) were studied. In all eligible studies, authors had assessed the accuracy and integrity of HRM assay for both first-line (isoniazid, rifampin, streptomycin, ethambutol, pyrazinamide, and MDR-TB) and second-line (particularly ofloxacine) treatments, using standard methods such as sequencing, MGIT 960, and proportional method (Table 1). In these studies, different primers were designed related to antibiotic resistance including *katG*, *mab-inhA*, *rpoB*, *rrs*, *rpsL*, *eis*, *embB*, *pncA*, *gyrA*‏, and *gyrB*. Quality assessments showed that most included studies had a low risk of bias, which confirmed the proportion of selected studies.

### Meta-analysis results

The results obtained from forest plots showed that the sensitivity and specificity of HRM were acceptable for distinguishing DR-TB strains from susceptible strains. According to statistical analysis, the sensitivity and specificity of DR-TB cases were 89% (95% CI: 88–90) and 95% (95% CI: 94–96), respectively. However, the *I*^*2*^ values for sensitivity and specificity were higher than 50%, which indicated a significant heterogeneity. The diagnostic accuracy of HRM was measured by SROC curve, so that the AUC and Q* values were 0.9754 and 0.9289, respectively.

### Subgroup analysis

Due to a significant heterogeneity in the estimated results, we performed subgroup analysis to discover the source of heterogeneity. Each of 47 studies independently had measured *Mtb* resistance to isoniazid, rifampin, ethambutol, streptomycin, pyrazinamide, fluoroquinolones MDR-TB, and first line. Parameters such as sensitivity, specificity, diagnostic ORs, and SROC for these antibiotics are listed in Table 2.

**Table 2:** Pooled means of sensitivity and specificity, DOR, and SROC for each antibiotics

Drugs	Sensitivity (95% CI)	Specificity (95% CI)	DOR (95% CI)	SROC	
				AUC	Q^*^
Isoniazid	93% (91–95)	98% (97–99)	459.85 (198.64–1064.55)	0.987	0.953
Rifampin	94% (92–95)	97% (95–98)	414.98 (182.7–942.6)	0.963	0.935
Ethambutol	82% (75–88)	87% (83–90)	69.50 (10.57–457.09)	0.728	0.794
Streptomycin	82% (77–87)	95% (93–97)	92.82 (49.36–174.55)	0.957	0.900
Pyrazinamide	72% (65–78)	84% (78–89)	16.11 (3.11–83.5)	0.845	0.777
MDR-TB	90% (86–93)	98% (95–99)	404.41 (87.02–1879.41)	0.989	0.956
fluoroquinolones	86% (78–92)	99% (97–100)	274.63 (83.71–901.02)	0.997	0.980
First-line	89% (88–90)	95% (94–96)	232.05 (121.50–443.21)	0.973	0.925

**Fig. 2:**
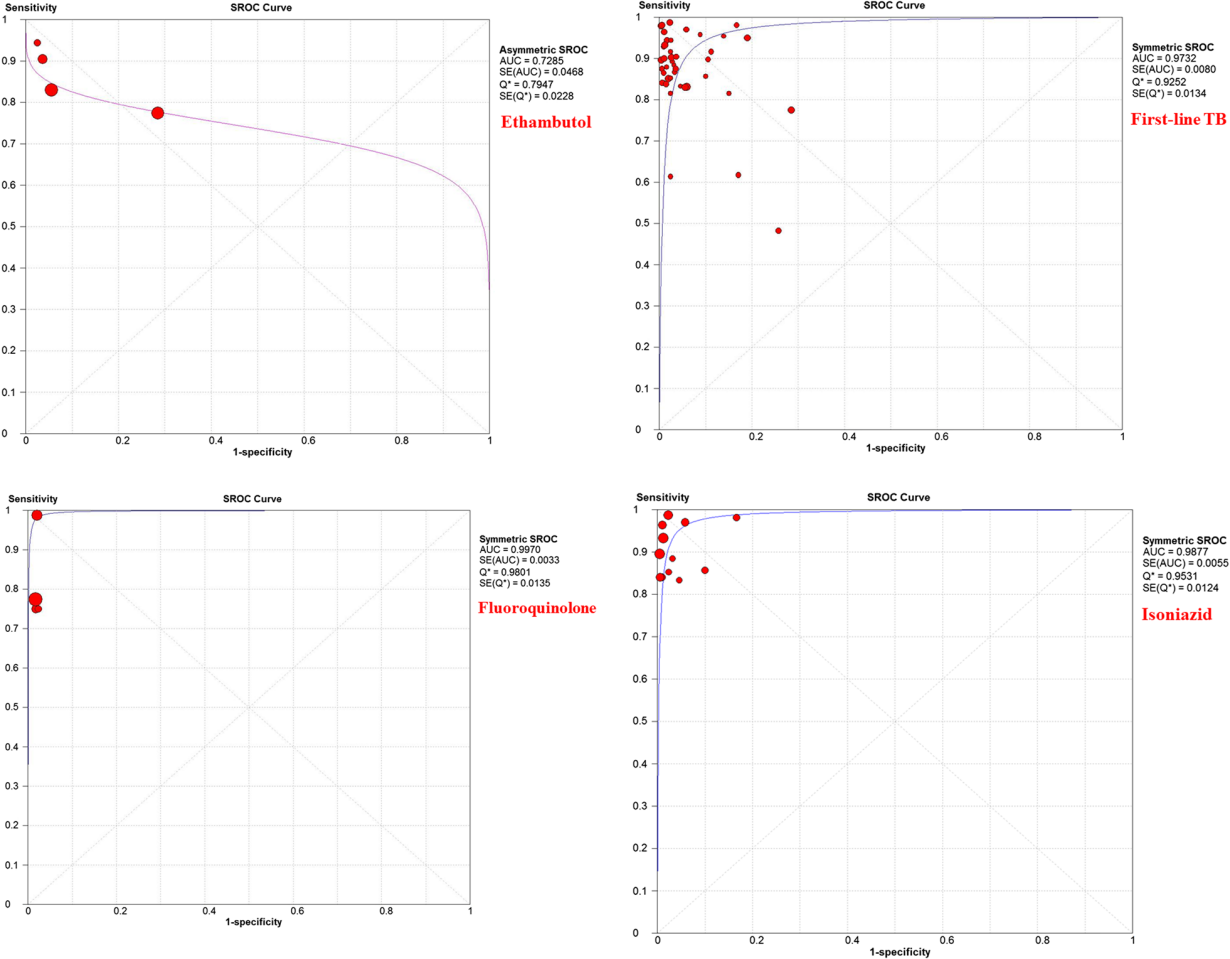

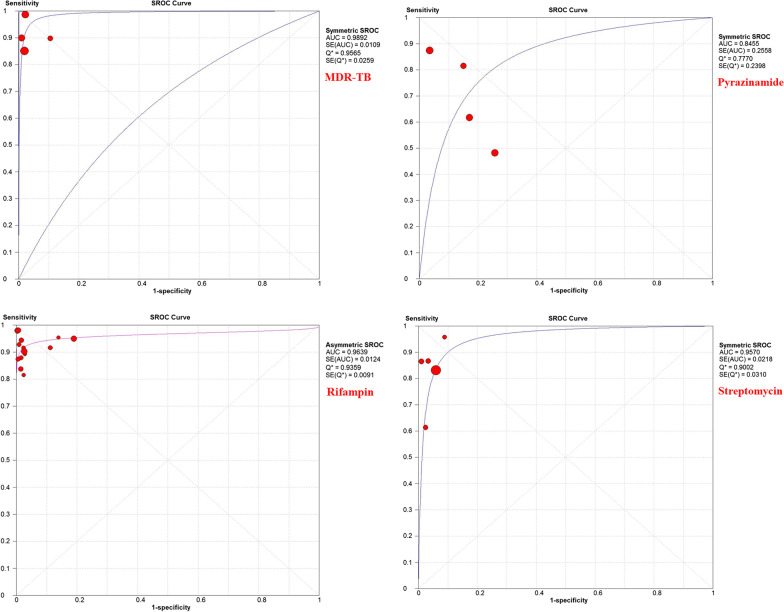
Summary Receiver Operating Characteristic curves of each antibiotic for discriminating drug-resistant TB cases (2.1: ethambutol, 2.2: first-line TB, 2.3: fluoroquinolone, 2.4: isoniazid, 2.5: MDR-TB, 2.6: pyrazinamide, 2.7: rifampin, and 2.8: streptomycin)

According to the results of subgrouping analysis, HRM is a reliable method for diagnosis of DR-TB cases, in particular the sensitivity and specificity for isoniazid, rifampin, and MDR-TB was higher than 90%. Furthermore, this method accurately discriminates resistance and susceptibility to other antibiotics such as ethambutol, streptomycin, pyrazinamide, and fluoroquinolones. The SROC curves represented the maximum polymerization spots of sensitivity and specificity associated with each of HRM assay (Fig. 2).

## Discussion

In recent years, the emergence of DR-TB strains is accounted a serious threat to TB control worldwide [45]. Diagnosis and screening of patients infected with MDR and extensively drug-resistant (XDR) strains is an important strategy to monitor and prevent the geographical spread of DR-TB strains [46]. Although conventional drug susceptibility testing such as proportional method, absolute concentration method, and resistance ratio method are known as reference methods, these are time-concuming and laborious [47]. To date, conventional drug susceptibility testing by liquid culture system has been introduced as colorimetric redox-indicator method, which in turn reduces the detection time to less than a week, but this system requires expensive tools and cannot be used in all developing countries [48, 49]. The Xpert MTB/RIF assay is a relatively new method recommended by WHO, but it should be noted that this method is only able to detect rifampin-resistant cases [50]. Among the molecular methods that can be used to determine TB antibiotic resistance, HRM is a fast, inexpensive, and simple method, which can detect different mutations using a small number of probes or even without a special probe [51]. In their meta-analysis study (using 7 articles), Yin et al. showed that the sensitivity and specificity of HRM in the diagnosing of rifampin-resistant TB cases were 94% and 99%, respectively [18]. A summary of common methods for detecting drug-resistant TB are listed in Table 3.

**Table 3:** Comparison of popular methods for the detection of drug-resistant tuberculosis

Test	Type	Advantage	Disadvantage	Limitation	Cost	Refs.
Culture-based methods	Solid-based media	High sensitivity and specificity	Cumbersome, time-consuming, laborious, need to standardization, need to skilled laboratory technicians, need to high-biosafety laboratories	Unreliability of HIV-positive cases, low sensitivity for extrapulmonary TB, less sensitive and slower than liquid culture	Relatively-expensive	[52, 53]
	Liquid-based media	Rapid automated, facilitate the processing of large numbers of specimens, high reproducibility, high sensitivity and specificity	Complexity, bio-safety concern, need for standardization, need for equipment	Inability to check the colony morphology of the growing bacteria, invisible contamination, overgrowth of NTM, need of expensive complex systems	High-cost	[54, 55]
	Colorimetric- based method	Rapid, inexpensive	Low sensitivity and specificity	NTM can produce cord factor, applied for culture isolates, isoniazid can lead to false-positive, need to large inoculum size	Low-cost	[56, 57]
Molecular-based methods	GeneXpert	A rapid, high reproducibility, high sensitivity and specificity, reliability of results for HIV-infected individuals, and extrapulmonary TB	Complexity, need for specialized laboratories, operator dependency	inability to detect isoniazid mono-resistance, mutation out of the *rpoB* was not detected, shelf life of the cartridges is only 18 months, very stable electricity supply is required, the instrument needs to be recalibrated annually	High-cost	[58, 59]
	Line probe assays	Detection of MTB complex, screening of resistance to isoniazid, rifampin, and MDR-TB, high sensitivity and specificity	Limited number of gene targets, high rate of uninterpretable results, risk of cross contamination due to its open-tube format	Applicable for smear-positive and culture isolates, time consuming due hybridization process, need to trained technicians	High-cost	[60, 61]
	HRM	Rapid, simple, closed-tube, homogenous, affordable method, cost-efficient	Variability of sensitivity and specificity for individual clinical diagnostic setting, misdiagnosis of small insertions and deletions, lack of databases	Poor accuracy in genotyping, safe amplicon length (more than 400 bp) depends on good PCR, instruments, and dyes	Low-cost	[18, 62–64]

To date, many studies have examined the accuracy of HRM for the diagnosis of DR-TB, however, no study has systematically evaluated the effectiveness of this method. In the present meta-analysis, we estimated the sensitivity and specificity of HRM in detecting DR-TB cases at 89% and 95%, respectively. According to our results, accuracy of HRM was slightly higher than other molecular methods, especially PCR-SSCP, while it was lower compared to the conventional drug susceptibility testing method. HRM method is credible method for detecting of mutations in specific genes; approximately 95% of rifampin-related mutations occur in the rifampicin resistance determining region (RRDR), while, 5% of those are outside this locus and cannot be detected by conventional molecular methods [65]. Nevertheless, some mutations such as nucleotide transversions (A:T and G:C) are difficult to distinguish, since they have very little influence on the overall thermal denaturation profile [66]. Sharma et al. showed that HRM could be a rapid and reliable method for the diagnosis of MDR-TB in cases of extrapulmonary TB [67]. In another study, Mu et al. examined this method for detecting DR-TB in formalin-fixed or paraffin‑embedded tissues; they found that the sensitivity of HRM for antibiotics such as rifampin, isoniazid, levofloxacin, and moxifloxacin was was 95.00%, 96.00%, 100%, and 100%, respectively, while its specificity was 95.15%, 95.92%, 94.69%, and 89.92%, respectively [68]. The stability of our findings are confirmed by similar studies and our results also showed that HRM is a trustworthy method for detecting resistance to some antibiotics, especially isoniazid and rifampin. In the present study, the heterogeneity was significant in some cases so that it was not eliminated by subgroup analysis; this issue requires a comprehensive program. This phenomenon may be due to differences in some factors such as study design, low sample size, references methods, examining tools, and dyes used. Despite its reliability, HRM has disadvatages including: (1) this method is performed only on cultured isolates; (2) HRM results are strongly depend on the quality of the extracted DNA; (3) researchers use a variety of protocols to set up HRM (self-design program). Our study had several limitations: (1) low sample size; (2) potential heterogeneity due to differences in study design and also unreliability of results; (3) inaccessibility to raw data for further analysis; (4) failure to assess the publication bias.

## Conclusion

Despite the limitations of the present study, according to previous studies, we have shown that HRM is an accurate method for diagnosing and monitoring DR-TB cases. This method, together with the results of drug susceptibility testing, seems to be a suitable strategy for rapid and inexpensive diagnosis of DR-TB cases.

## Data Availability

All data generated or analyzed during this study are included in this published article.

## Abbreviations

TB: Tuberculosis
DR-TB: Drug-resistant tuberculosis
HRM: High resolution melting
*Mtb*: *Mycobacterium tuberculosis*
SROC: Summary receiver operating characteristic
AUC: Curve and the area under the curve
TP: True positive
FP: False positive
TN: True negative
FN: False negative
NOS: Newcastle–Ottawa Scale
PRISMA: Preferred reporting items for systematic reviews and meta-analysis
SNP: Single nucleotide polymorphism

## References

[1] Drain PK, Bajema KL, Dowdy D, Dheda K, Naidoo K, Schumacher SG, Ma S, Meermeier E, Lewinsohn DM, Sherman DR (2018). Incipient and subclinical tuberculosis: a clinical review of early stages and progression of infection. Clin Microbiol Rev.

[2] Keikha M, Esfahani BN (2018). The relationship between tuberculosis and lung cancer. Adv Biomed Res.

[3] Keikha M, Karbalaei M (2021). Overview on coinfection of HTLV-1 and tuberculosis: Mini-review. J Clin Tuberc Other Mycobact Dis.

[4] Organization WH: Global tuberculosis report 2020: executive summary. 2020.

[5] Keikha M (2020). There is significant relationship between Beijing genotype family strains and resistance to the first-line anti-tuberculosis drugs in the Iranian population. J Clin Tuberc Other Mycobact Dis.

[6] Weyer K, Dennis Falzon D, Jaramillo E, Zignol M, Mirzayev F, Raviglione M (2017). Drug-resistant tuberculosis: what is the situation, what are the needs to roll it back. AMR control.

[7] Keikha M, Karbalaei M (2019). Antithetical effects of MicroRNA molecules in tuberculosis pathogenesis. Adv Biomed Res.

[8] Migliori GB, Tiberi S, Zumla A, Petersen E, Chakaya JM, Wejse C, Torrico MM, Duarte R, Alffenaar JW, Schaaf HS (2020). MDR/XDR-TB management of patients and contacts: challenges facing the new decade The 2020 clinical update by the Global Tuberculosis Network. Int J Infect Dis.

[9] Organization WH (2007). The global MDR-TB & XDR-TB response plan 2007–2008.

[10] Jacobson KR, Theron D, Kendall EA, Franke MF, Barnard M, Van Helden PD, Victor TC, Streicher EM, Murray MB, Warren RM (2013). Implementation of GenoType MTBDR plus reduces time to multidrug-resistant tuberculosis therapy initiation in South Africa. Clin Infect Dis.

[11] Sohn H, Aero AD, Menzies D, Behr M, Schwartzman K, Alvarez GG, Dan A, McIntosh F, Pai M, Denkinger CM (2014). Xpert MTB/RIF testing in a low tuberculosis incidence, high-resource setting: limitations in accuracy and clinical impact. Clin Infect Dis.

[12] Bemer P, Palicova F, Rüsch-Gerdes S, Drugeon HB, Pfyffer GE (2002). Multicenter evaluation of fully automated BACTEC Mycobacteria Growth Indicator Tube 960 system for susceptibility testing of *Mycobacterium tuberculosis*. J Clin Microbiol.

[13] Fluit AC, Visser MR, Schmitz F-J (2001). Molecular detection of antimicrobial resistance. Clin Microbiol Rev.

[14] Krypuy M, Newnham GM, Thomas DM, Conron M, Dobrovic A (2006). High resolution melting analysis for the rapid and sensitive detection of mutations in clinical samples: KRAS codon 12 and 13 mutations in non-small cell lung cancer. BMC Cancer.

[15] Liew M, Nelson L, Margraf R, Mitchell S, Erali M, Mao R, Lyon E, Wittwer C (2006). Genotyping of human platelet antigens 1 to 6 and 15 by high-resolution amplicon melting and conventional hybridization probes. J Mol Diagn.

[16] Athamanolap P, Parekh V, Fraley SI, Agarwal V, Shin DJ, Jacobs MA, Wang T-H, Yang S (2014). Trainable high resolution melt curve machine learning classifier for large-scale reliable genotyping of sequence variants. PLoS ONE.

[17] Montgomery JL, Sanford LN, Wittwer CT (2010). High-resolution DNA melting analysis in clinical research and diagnostics. Expert Rev Mol Diagn.

[18] Yin X, Zheng L, Liu Q, Lin L, Hu X, Hu Y, Wang Q (2013). High-resolution melting curve analysis for rapid detection of rifampin resistance in *Mycobacterium tuberculosis*: a meta-analysis. J Clin Microbiol.

[19] Moher D, Shamseer L, Clarke M, Ghersi D, Liberati A, Petticrew M, Shekelle P, Stewart LA (2015). Preferred reporting items for systematic review and meta-analysis protocols (PRISMA-P) 2015 statement. Syst Rev.

[20] Pietzka AT, Indra A, Stöger A, Zeinzinger J, Konrad M, Hasenberger P, Allerberger F, Ruppitsch W (2009). Rapid identification of multidrug-resistant *Mycobacterium tuberculosis* isolates by rpoB gene scanning using high-resolution melting curve PCR analysis. J Antimicrob Chemother.

[21] Choi GE, Lee SM, Yi J, Hwang SH, Kim HH, Lee EY, Cho EH, Kim JH, Kim H-J, Chang CL (2010). High-resolution melting curve analysis for rapid detection of rifampin and isoniazid resistance in *Mycobacterium tuberculosis* clinical isolates. J Clin Microbiol.

[22] Ong DC, Yam W-C, Siu GK, Lee AS (2010). Rapid detection of rifampicin-and isoniazid-resistant *Mycobacterium tuberculosis* by high-resolution melting analysis. J Clin Microbiol.

[23] Ramirez MV, Cowart KC, Campbell PJ, Morlock GP, Sikes D, Winchell JM, Posey JE (2010). Rapid detection of multidrug-resistant *Mycobacterium tuberculosis* by use of real-time PCR and high-resolution melt analysis. J Clin Microbiol.

[24] Wang F, Shen H, Guan M, Wang Y, Feng Y, Weng X, Wang H, Zhang W (2011). High-resolution melting facilitates mutation screening of rpsL gene associated with streptomycin resistance in *Mycobacterium tuberculosis*. Microbiol Res.

[25] Chen X, Kong F, Wang Q, Li C, Zhang J, Gilbert GL (2011). Rapid detection of isoniazid, rifampin, and ofloxacin resistance in *Mycobacterium tuberculosis* clinical isolates using high-resolution melting analysis. J Clin Microbiol.

[26] Lee AS, Ong DC, Wong JC, Siu GK, Yam W-C (2012). High-resolution melting analysis for the rapid detection of fluoroquinolone and streptomycin resistance in *Mycobacterium tuberculosis*. PLoS ONE.

[27] Yadav R, Sethi S, Mewara A, Dhatwalia S, Gupta D, Sharma M (2012). Rapid detection of rifampicin, isoniazid and streptomycin resistance in M ycobacterium tuberculosis clinical isolates by high-resolution melting curve analysis. J Appl Microbiol.

[28] Nagai Y, Iwade Y, Hayakawa E, Nakano M, Sakai T, Mitarai S, Katayama M, Nosaka T, Yamaguchi T (2013). High resolution melting curve assay for rapid detection of drug-resistant *Mycobacterium tuberculosis*. J Infect Chemother.

[29] Nour MS, El-Shokry MH, Shehata IH, Aziz A (2013). Evaluation of rezasurin microtiter assay and high resolution melting curve analysis for detection of rifampicin and isoniazid resistance of *Mycobacterium tuberculosis* clinical isolates. Clin Lab.

[30] Haeili M, Fooladi A, Bostanabad S, Sarokhalil D, Siavoshi F, Feizabadi M (2014). Rapid screening of rpoB and katG mutations in *Mycobacterium tuberculosis* isolates by high-resolution melting curve analysis. Indian J Med Microbiol.

[31] Pholwat S, Stroup S, Gratz J, Trangan V, Foongladda S, Kumburu H, Juma SP, Kibiki G, Houpt E (2014). Pyrazinamide susceptibility testing of *Mycobacterium tuberculosis* by high resolution melt analysis. Tuberculosis.

[32] Malhotra B, Goyal S, Bhargava S, Reddy P, Chauhan A, Tiwari J (2015). Rapid detection of rifampicin resistance in *Mycobacterium tuberculosis* by high-resolution melting curve analysis. Int J Tuberc Lung Dis.

[33] Pholwat S, Liu J, Stroup S, Gratz J, Banu S, Rahman SM, Ferdous SS, Foongladda S, Boonlert D, Ogarkov O (2015). Integrated microfluidic card with TaqMan probes and high-resolution melt analysis to detect tuberculosis drug resistance mutations across 10 genes. MBio.

[34] Osman F, Ismail F, Osman A, Omar S, Said H, Ismail N (2016). High resolution melting curve analysis for rapid detection of pyrazinamide resistance in *Mycobacterium tuberculosis* clinical isolates. J Tuberc Res.

[35] Galarza M, Fasabi M, Levano KS, Castillo E, Barreda N, Rodriguez M, Guio H (2016). High-resolution melting analysis for molecular detection of multidrug resistance tuberculosis in Peruvian isolates. BMC Infect Dis.

[36] Anthwal D, Gupta RK, Bhalla M, Bhatnagar S, Tyagi JS, Haldar S (2017). Direct detection of rifampin and isoniazid resistance in sputum samples from tuberculosis patients by high-resolution melt curve analysis. J Clin Microbiol.

[37] Rezaei F, Haeili M, Fooladi AI, Feizabadi MM (2017). High resolution melting curve analysis for rapid detection of streptomycin and ethambutol resistance in *Mycobacterium tuberculosis*. Maedica.

[38] Sirous M, Khosravi AD, Tabandeh MR, Salmanzadeh S, Ahmadkhosravi N, Amini S (1819). Molecular detection of rifampin, isoniazid, and ofloxacin resistance in Iranian isolates of *Mycobacterium tuberculosis* by high-resolution melting analysis. Infect Drug Resist.

[39] Negi SS, Singh P, Bhargava A, Chandrakar S, Gaikwad U, Das P, Behra A (2018). Effective pragmatic approach of diagnosis of multidrug-resistant tuberculosis by high-resolution melt curve assay. Int J Mycobacteriol.

[40] Filipenko M, Dymova M, Cherednichenko A, Khrapov E, Mishukova O, Schwartz YS (2019). Detection of Mutations in *Mycobacterium tuberculosis* pncA gene by modified high-resolution melting curve analysis of PCR products. Bull Exp Biol Med.

[41] Arefzadeh S, Azimi T, Nasiri MJ, Nikpor Z, Dabiri H, Doustdar F, Goudarzi H, Allahyartorkaman M (2020). High-resolution melt curve analysis for rapid detection of rifampicin resistance in *Mycobacterium tuberculosis*: a single-centre study in Iran. New Microb New Infect.

[42] Anukool U, Phunpae P, Tharinjaroen CS, Butr-Indr B, Saikaew S, Netirat N, Intorasoot S, Suthachai V, Tragoolpua K, Chaiprasert A (2020). Genotypic distribution and a potential diagnostic assay of multidrug-resistant tuberculosis in Northern Thailand. Infect Drug Resist.

[43] Wang J, Zhao W, Liu R, Huo F, Dong L, Xue Y, Wang Y, Xue Z, Ma L, Pang Y (2020). Rapid detection of ethambutol-resistant *Mycobacterium tuberculosis* from sputum by high-resolution melting analysis in Beijing China. Infect Drug Resist.

[44] Keikha M, Karbalaei M: P2X7 polymorphism (rs3751143) and its reliability as a diagnostic biomarker for tuberculosis: a systematic review and meta-analysis. *Indian J Tuberc* 2021. 10.1016/j.ijtb.2021.04.004 35074157

[45] Keikha M, Eslami M, Yousefi B, Karbalaei M (2020). Overview of multistage subunit tuberculosis vaccines: advantages and challenges. Reviews in Medical Microbiology.

[46] Dey T, Brigden G, Cox H, Shubber Z, Cooke G, Ford N (2013). Outcomes of clofazimine for the treatment of drug-resistant tuberculosis: a systematic review and meta-analysis. J Antimicrob Chemother.

[47] Van Deun A, Martin A, Palomino JC: Diagnosis of drug-resistant tuberculosis: reliability and rapidity of detection [State of the art series. Drug-resistant tuberculosis. Edited by CY. Chiang. Number 3 in the series]. *Int J Tuberc Lung Dis *2010, 14(2):131–140. 20074402

[48] Pai M, Kalantri S, Pascopella L, Riley LW, Reingold AL (2005). Bacteriophage-based assays for the rapid detection of rifampicin resistance in *Mycobacterium tuberculosis*: a meta-analysis. J Infect.

[49] Martin A, Portaels F, Palomino JC (2007). Colorimetric redox-indicator methods for the rapid detection of multidrug resistance in *Mycobacterium tuberculosis*: a systematic review and meta-analysis. J Antimicrob Chemother.

[50] Blakemore R, Story E, Helb D, Kop J, Banada P, Owens MR, Chakravorty S, Jones M, Alland D (2010). Evaluation of the analytical performance of the Xpert MTB/RIF assay. J Clin Microbiol.

[51] Erali M, Wittwer CT (2010). High resolution melting analysis for gene scanning. Methods.

[52] Kalokhe AS, Lee JC, Ray SM, Anderson AM, Nguyen MLT, Wang YF, Shafiq M, Metchock B (2013). Multidrug-resistant tuberculosis drug susceptibility and molecular diagnostic testing. Am J Med Sci.

[53] van Klingeren B, Dessens-Kroon M, van der Laan T, Kremer K, van Soolingen D (2007). Drug susceptibility testing of *Mycobacterium tuberculosis* complex by use of a high-throughput, reproducible, absolute concentration method. J Clin Microbiol.

[54] Lawson L, Emenyonu N, Abdurrahman ST, Lawson JO, Uzoewulu GN, Sogaolu OM, Ebisike JN, Parry CM, Yassin MA, Cuevas LE (2013). Comparison of *Mycobacterium tuberculosis* drug susceptibility using solid and liquid culture in Nigeria. BMC Res Notes.

[55] Nguyen TNA, Berre A-L, Bañuls A-L, Nguyen TVA (2019). Molecular diagnosis of drug-resistant tuberculosis; a literature review. Front Microbiol.

[56] Alcántara R, Fuentes P, Antiparra R, Santos M, Gilman RH, Kirwan DE, Zimic M, Sheen P (2019). MODS-Wayne, a colorimetric adaptation of the Microscopic-Observation Drug Susceptibility (MODS) assay for detection of *Mycobacterium tuberculosis* pyrazinamide resistance from sputum samples. J Clin Microbiol.

[57] Syre H, Phyu S, Sandven P, Bjorvatn B, Grewal H (2003). Rapid colorimetric method for testing susceptibility of *Mycobacterium tuberculosis* to isoniazid and rifampin in liquid cultures. J Clin Microbiol.

[58] Evans CA (2011). GeneXpert—a game-changer for tuberculosis control?. PLoS Med.

[59] Mechal Y, Benaissa E, Benlahlou Y, Bssaibis F, Zegmout A, Chadli M, Malik YS, Touil N, Abid A, Maleb A (2019). Evaluation of GeneXpert MTB/RIF system performances in the diagnosis of extrapulmonary tuberculosis. BMC Infect Dis.

[60] Ruesch-Gerdes S, Ismail N, Denkinger C, Gilpin C, Tahirli R, van Deun A, Rigouts L, Hillemann D: REPORT FOR WHO Non-inferiority Evaluation of Nipro NTM+ MDRTB and Hain GenoType MTBDRplus V2 Line Probe Assays. In*.*: Foundation for Innovative New Diagnostics Geneva, Switzerland; 2015.

[61] Ling DI, Zwerling AA, Pai M (2008). GenoType MTBDR assays for the diagnosis of multidrug-resistant tuberculosis: a meta-analysis. Eur Respir J.

[62] Wittwer CT (2009). High-resolution DNA melting analysis: advancements and limitations. Hum Mutat.

[63] Słomka M, Sobalska-Kwapis M, Wachulec M, Bartosz G, Strapagiel D (2017). High resolution melting (HRM) for high-throughput genotyping—limitations and caveats in practical case studies. Int J Mol Sci.

[64] Li B-S, Wang X-Y, Ma F-L, Jiang B, Song X-X, Xu A-G (2011). Is high resolution melting analysis (HRMA) accurate for detection of human disease-associated mutations? A meta analysis. PLoS ONE.

[65] Caws M, Drobniewski F (2001). Molecular techniques in the diagnosis of *Mycobacterium tuberculosis* and the detection of drug resistance. Ann N Y Acad Sci.

[66] Hoek K, van Pittius NG, Moolman-Smook H, Carelse-Tofa K, Jordaan A, Van Der Spuy G, Streicher E, Victor T, Van Helden P, Warren R (2008). Fluorometric assay for testing rifampin susceptibility of *Mycobacterium tuberculosis* complex. J Clin Microbiol.

[67] Sharma K, Sharma M, Singh S, Modi M, Sharma A, Ray P, Varma S (2017). Real-time PCR followed by high-resolution melting curve analysis: a rapid and pragmatic approach for screening of multidrug-resistant extrapulmonary tuberculosis. Tuberculosis.

[68] Mu J, Liu Z, Zhang C, Wang C, Du W, Lin H, Li K, Song J, Che N, Liu H (2021). Performance of the MeltPro MTB assays in the diagnosis of drug-resistant tuberculosis using formalin-fixed paraffin-embedded tissues. Am J Clin Pathol.

